# siRNA-Mediated Silencing of *doublesex* during Female Development of the Dengue Vector Mosquito *Aedes aegypti*

**DOI:** 10.1371/journal.pntd.0004213

**Published:** 2015-11-06

**Authors:** Keshava Mysore, Longhua Sun, Michael Tomchaney, Gwyneth Sullivan, Haley Adams, Andres S. Piscoya, David W. Severson, Zainulabeuddin Syed, Molly Duman-Scheel

**Affiliations:** 1 Department of Medical and Molecular Genetics, Indiana University School of Medicine, South Bend, Indiana, United States of America; 2 Eck Institute for Global Health, University of Notre Dame, Notre Dame, Indiana, United States of America; 3 Department of Biological Sciences, University of Notre Dame, Notre Dame, Indiana, United States of America; University of California, Irvine, UNITED STATES

## Abstract

The development of sex-specific traits, including the female-specific ability to bite humans and vector disease, is critical for vector mosquito reproduction and pathogen transmission. Doublesex (Dsx), a terminal transcription factor in the sex determination pathway, is known to regulate sex-specific gene expression during development of the dengue fever vector mosquito *Aedes aegypti*. Here, the effects of developmental siRNA-mediated *dsx* silencing were assessed in adult females. Targeting of *dsx* during *A*. *aegypti* development resulted in decreased female wing size, a correlate for body size, which is typically larger in females. siRNA-mediated targeting of *dsx* also resulted in decreased length of the adult female proboscis. Although *dsx* silencing did not impact female membrane blood feeding or mating behavior in the laboratory, decreased fecundity and fertility correlated with decreased ovary length, ovariole length, and ovariole number in *dsx* knockdown females. Dsx silencing also resulted in disruption of olfactory system development, as evidenced by reduced length of the female antenna and maxillary palp and the sensilla present on these structures, as well as disrupted odorant receptor expression. Female lifespan, a critical component of the ability of *A*. *aegypti* to transmit pathogens, was also significantly reduced in adult females following developmental targeting of *dsx*. The results of this investigation demonstrate that silencing of *dsx* during *A*. *aegypti* development disrupts multiple sex-specific morphological, physiological, and behavioral traits of adult females, a number of which are directly or indirectly linked to mosquito reproduction and pathogen transmission. Moreover, the olfactory phenotypes observed connect Dsx to development of the olfactory system, suggesting tha*t A*. *aegypti* will be an excellent system in which to further assess the developmental genetics of sex-specific chemosensation.

## Author Summary

Only adult female mosquitoes, which require blood meals for reproduction, bite humans and spread diseases. The genes that regulate development of sex-specific traits may therefore represent novel targets for mosquito control. Here, we examine the effects of silencing the sex-determination gene *doublesex (dsx)* during development of the human disease vector mosquito *Aedes aegypti*. Targeting of *dsx* resulted in decreased length of the female wing and proboscis, ovary and reproductive defects, and disruption of olfactory system development. Female lifespan, a critical aspect of mosquito pathogen transmission, was also significantly reduced in adult females following developmental targeting of *dsx*. The results of this investigation demonstrate that silencing of *dsx* during *A*. *aegypti* development disrupts multiple sex-specific morphological, physiological, and behavioral traits of adult females, a number of which are directly or indirectly linked to mosquito reproduction and pathogen transmission. The results obtained also connect Dsx to development of the mosquito olfactory system, suggesting tha*t A*. *aegypti* will be an excellent system in which to further assess the developmental genetics of sex-specific chemosensation.

## Introduction

Most animal species display sexually dimorphic behaviors, the majority of which are linked to sexual reproduction [1]. Disease vector mosquitoes are excellent subjects for studies that explore the biological basis of sexual dimorphism. Only adult female mosquitoes, which require blood meals for reproduction, bite humans and transmit pathogens. Females differ from males in morphological, physiological, and behavioral traits that are critical components of their ability to spread diseases, including feeding behaviors, longevity, and susceptibility to infections. Researchers have therefore had a long-standing interest in the potential to manipulate genetic components of the sex determination pathway and sexual differentiation for vector control. Moreover, success of the sterile insect technique (SIT) and other genetic strategies designed to eliminate large populations of mosquitoes is dependent upon efficient sex-sorting of males and females, and many have argued that such sex-sorting, as well as insect sterilization itself, is best achieved through large-scale genetic or transgenic approaches (reviewed by [2, 3]). Although the genes that regulate sex-specification and development of mosquito sexual dimorphism may represent novel targets for vector control, most of these genes have not yet been functionally characterized in vector mosquitoes.

Research in *Drosophila melanogaster* identified a mutation in the *doublesex (dsx)* gene that transformed males and females into intersexes [4]. Subsequent molecular analyses demonstrated that the *dsx* gene encodes a key terminal transcription factor in the sex-determination pathway that controls *Drosophila* male and female sexual differentiation [5–7]. *Drosophila dsx* pre-mRNAs are spliced in a sex-specific manner [8, 9], generating male (Dsx^M^) and female (Dsx^F^) proteins with a common N-terminus and DNA-binding domain, but distinct male and female C-termini that differentially regulate sex-specific gene expression (reviewed by [10, 11]). Studies in diverse insects have demonstrated that although primary signals for sex determination vary within the insect order [12], all relay their signal through the sex-specific splicing of *dsx*, which plays a well-conserved role as a transcription factor that regulates expression of downstream target genes which contribute to sexual differentiation [13–25]. The roles of Dsx have been particularly well studied in a variety of beetle species [17, 18, 19, 21, 26]. For example, sex-specific Dsx splice forms are known to regulate sexually dimorphic exaggerated horn development in two species of beetles, *Onthophagus taurus* [18] and the rhinoceros beetle *Trypoxylus dichotomus* [19]. Dsx function was also characterized in the red flour beetle *Tribolium castaneum*, in which it is required for oocyte development, egg production, and egg hatching [17]. More recently, Dsx was shown to regulate sexually dimorphic mandible development in the stag beetle *Cyclommatus metallifer* [21]. Here, we examine the function of *dsx* during development of the disease vector mosquito *Aedes aegypti*, which exhibits innate sexually dimorphic behaviors that contribute to the transmission of dengue, yellow fever, and chikungunya viruses [27].

Salvemini et al. [28] detected male *(dsx* ^*M*^ *)* and female (*dsx* ^*F*^ *)* splice variants of *dsx* in *A*. *aegypti*. Recently, Hall et al. [29] described characterization of a male-determining locus (M-locus) gene, *Nix*, a male-determining factor (M factor) in *A*. *aegypti* that is required and sufficient to initiate male development, and which encodes a potential splicing factor. The absence of *Nix* shifts the alternative splicing of *dsx* toward the female-specific *dsx* ^*F*^ splice form, suggesting that *Nix* normally promotes splicing of *dsx* ^*M*^. Although the sex-specific *dsx* splice forms likely direct sexually dimorphic mosquito development, functional analysis of *dsx* in *A*. *aegypti* is lacking. In a recent study from our laboratory [30], we detected sex-specific *dsx* expression in the pupal brain suggesting that sexually dimorphic neural development in *A*. *aegypti* may require *dsx* function. In support of this, a search of the *A*. *aegypti* genome sequence uncovered 732 Dsx consensus binding sites, 48 of which flank dimorphically expressed genes identified in male vs. female pupal head transcriptome microarray experiments [30]. *A*. *aegypti* genes flanked by Dsx consensus binding sites group under a number of significant gene ontology terms, many of which are linked to neurological processes or neural development, suggesting that Dsx may regulate sex-specific gene expression in the developing mosquito brain. To examine this, we [30] used small interfering RNA (siRNA)-mediated gene targeting to silence *dsx* during *A*. *aegypti* pupal development. In our study, siRNAs corresponding to different target sequences in exon 2, which is common to male and female splice variants, were injected into *A*. *aegypti* pupae. These targeting experiments demonstrated that Dsx is required for the regulation of sex-specific gene expression during *A*. *aegypti* neural development. The results of our initial investigation [30], in conjunction with studies performed in a variety of insects (reviewed by [24]), support the hypothesis that Dsx regulates the development of sex-specific characters in *A*. *aegypti*. Here we test this hypothesis by examining the impact of larval and pupal *dsx* silencing on the development of sex-specific traits in adult female mosquitoes.

Whyard et al. [31] recently used RNA interference (RNAi) to target the female-specific isoform of *A*. *aegypti dsx (dsx* ^*F*^ *)* during development. Their *dsx* targeting protocol differed from that which we used [30] in that they used longer pieces of dsRNA (as opposed to siRNA) targeting the two female-specific *dsx* exons (as opposed to exon two, which is common) that was delivered by soaking mosquito larvae in dsRNA or by feeding the larvae *E*. *coli* expressing *dsx*-targeting dsRNA (rather than through microinjection). Although the Whyard et al. [31] *dsx* targeting strategies resulted in highly male-biased populations of mosquitoes, the number of *dsx* dsRNA-treated larvae that developed into adults was halved relative to the negative controls, and with no significant increase in the number of males observed, suggesting that the majority of females simply failed to survive to adulthood, which did not permit analysis of sex-specific characters in these animals. Our ability to analyze late pupae microinjected with siRNAs targeting a separate region of the *dsx* gene (exon two) suggested that this targeting strategy [30], which differs from the Whyard et al. [31] procedure as noted above, may facilitate analysis of adult female phenotypes. Such analyses are of great interest given that adult females are responsible for transmission of pathogens that result in human diseases. Indeed, we found that use of a pupal microinjection procedure to deliver siRNA targeting exon 2 [30], as well as the use of chitosan nanoparticles [32–34] to deliver these same exon 2 targeting siRNAs to *A*. *aegypti* larvae, silenced *dsx* while permitting female survival. Use of these targeting strategies allowed us to examine adult female morphological, physiological, and behavioral phenotypes that result from developmental silencing of *dsx*.

## Materials and Methods

### Animal rearing and sexing

The *A*. *aegypti* Liverpool-IB12 (LVP-IB12) strain (from D.W. Severson, Notre Dame, IN), from which the genome sequence [35] was generated, was used in this investigation. The mosquitoes were reared as described [36], except that an artificial membrane sheep blood (HemoStat Laboratories, Dixon, CA) feeding system was utilized. Mosquitoes were maintained in an insectary at 26°C, at ~80% humidity, and under a 12 hr light/12 hr dark cycle with 1 hr crepuscular periods at the beginning and end of each light cycle. Mosquito larvae were fed on a suspension of dried beef liver powder, and adults were provided cotton soaked with 10% sugar solution. Pupae were sexed on the basis of differing pupal tail morphology as described by Christophers [37]. Adults were sexed on the basis of external characters [37]. Sexes of *dsx*-silenced adults were further confirmed in a subset of animals through dissection to assess the presence of testes or ovaries.

### Whole mount *in situ* hybridization and immunohistochemistry

*In situ* hybridization was performed as previously described [38]. Riboprobes corresponding to the following genes were synthesized according to the Patel [39] protocol: *OR 2 (AAEL005999)*, *OR 9 (AAEL006005)*, *OR 62 (AAEL011796)*, and *OR 123 (AAEL017537)* and *dsx* (*AAEL009114;* probe corresponded to exon 2 which is common to males and females). At least 20 tissue specimens were processed for each *in situ* hybridization experiment, and at least two replicate experiments were performed. A sense riboprobe was used as a control in all hybridization experiments. Immunohistochemical staining was performed as described previously [33, 40] using Texas Red-X Phalloidin and TO-PRO-3 iodide, both which were obtained from Molecular Probes (Eugene, OR). Following processing, the tissues were mounted and imaged on a Zeiss 710 confocal microscope using Zen software, and images were analyzed with FIJI ImageJ and Adobe Photoshop CC 2014 software. For *OR* transcript analyses, mean gray values (average signal intensity over the selected area) were calculated for digoxigenin-labeled *OR* transcript signal in 25 control or experimental antennae combined from two replicate experiments. Data were statistically analyzed using one-way ANOVA followed by the Bonferroni post hoc test.

### RNAi experiments

Targeting of *dsx* (*AAEL009114)* was performed as described previously [30]. Two siRNAs, *dsx-KD A* and *dsx-KD B*, that correspond to different target sequences in exon 2, which is common to both the male and female *dsx* splice variants, were used in *dsx* silencing experiments. The sequences of these siRNA duplexes, which were purchased from Integrated DNA Technology (IDT, Coralville, IA) and confirmed through BLAST searches to have no significant homology to *A*. *aegypti* genes other than *dsx*, are as follows:

*Dsx-KD A*: 5’ rCrArGrGrArArCrArGrArCrGrArCrGrArArCrUrUrGrUrCAA3’ / 5’rUrUrGrArCrArArGrUrUrCrGrUrCrGrUrCrUrGrUrUrCrCrUrGrArG3’, and *Dsx-KD B*: 5’rCrArArGrArUrCrGrCrUrGrGrArUrGrGrUrArArArGrArUGT3’ / 5’rArCrArUrCrUrUrUrArCrCrArUrCrCrArGrCrGrArUrCrUrUrGrCrG3’. All phenotypes were confirmed following knockdown (KD) with both *dsx-KD A* and *dsx-KD B*, suggesting that none of the phenotypes reported herein were the result of off-site targeting by either siRNA. A scrambled version of *dsx KD B*, an siRNA duplex lacking significant sequence homology to any genes in the *A*. *aegypti* genome, was used for control experiments:

5’rGrArArGrArGrCrArCrUrGrArUrArGrArUrGrUrUrArGrCGT3’ / 5’rArCrGrCrUrArArCrArUrCrUrArUrCrArGrUrGrCrUrCrUrUrCrCrG3’. None of the phenotypes reported were observed in control-injected animals, which were not significantly different than wild type animals for any of the phenotypes assessed.

siRNA was microinjected into pupae [30] as described previously. Chitosan/siRNA-mediated targeting of *dsx* was performed using the procedure described by Mysore et al. [33], which was adapted from Zhang et al. [32] and is described in detail in Zhang et al. [34]. Silencing of *dsx* was confirmed through *in situ* hybridization as discussed in the recent siRNA-mediated *dsx* gene targeting study [30]. To quantify knockdown levels, mean gray values were calculated as described [41] for digoxigenin-labeled *dsx* transcript signal in brains and antennae from minimally 20 control or experimental specimens combined from two separate replicate experiments. These data were statistically analyzed using one-way ANOVA followed by the Bonferroni post hoc test.

### Analysis of morphological characters

Wing length and area, proboscis, antenna, and maxillary palp lengths were assessed in females following chitosan/siRNA nanoparticle mediated targeting of *dsx* as described above. For these experiments, structures were dissected from sugar-fed ~10 day old adult female mosquitoes, mounted and analyzed with a Zeiss Axioimager equipped with a Spot Flex camera. Areas and lengths were measured using Fiji Image J software. Wing lengths were measured from the apical notch to the axillary margin, excluding the wing fringe as described in [37]. To minimize measurement errors, all appendage measurements were determined by a single researcher. Data from at least four replicate experiments were combined for statistical comparisons, which were performed using Graphpad Prism 6 software with one-way ANOVA followed by the Bonferroni post hoc test. Maxillary palp and antennal sensillary morphology was further assessed (following pupal microinjection of siRNA) through scanning electron microscopy (SEM) with an FEI-MAGELLAN 400 FESEM as previously described [42]. Briefly, female heads were placed in acetone for 24 hrs and subjected to critical point drying followed by sputter coating with gold/Iridium. Control vs. *dsx*-KD olfactory structures were visualized under SEM and assessed for numerical and structural anomalies. Data from replicate experiments were combined and statistically analyzed with one-way ANOVA followed by the Bonferroni post hoc test.

### Blood feeding, reproductive, and survival assays

Blood feeding behavior was visually assessed through analysis of engorged female abdomens following plasma membrane blood feedings which were one hour in duration. The number of eggs produced per female (fecundity) and eggs produced per female that generated first instar larvae (fertility) were assessed as described by Hill et al. [43]. The number of fertile females, which served as evidence of successful mating, was also recorded. Survivorship, which was monitored in individual females following completion of the fecundity assays, was performed and analyzed as described by Hill et al. [43]. Ovary length was assessed in four day-old adults prior to blood feeding, as well as in 10 day post blood-fed females. The number of follicles was assessed in four day-old adults prior to blood feeding, while ovariole number and length was assessed five days post blood feeding. For these assays, ovaries were immunohistochemically processed as described above, mounted, and then analyzed with a Zeiss 710 confocal microscope using Zen software. Scanned images were analyzed using FIJI and Adobe Photoshop CC 2014 software. With the exception of lifespan, which was analyzed using Kaplan-Meier survival curves, data were analyzed using Graphpad Prism 6 software with one-way ANOVA followed by the Bonferroni post hoc test.

### Sequence analyses

Introns as well as 5’ flanking sequences 0–5 kb upstream of the open reading frames of *A*. *aegypti OR* genes were exported from VectorBase [44]. These sequences were searched (using ClustalW) for the Clough et al. [45] Dsx consensus binding site sequence: VHHACWAWGWHDN. Sequences with no more than one mismatch are reported.

### Gene identification numbers

The following genes were studied in this investigation: *dsx* (*AAEL009114)*, *OR 2 (AAEL005999)*, *OR 9 (AAEL006005)*, *OR 62 (AAEL011796)*, and *OR 123 (AAEL017537)*.

## Results

### siRNA-mediated silencing of *dsx* during larval and pupal *A*. *aegypti* development

siRNA-mediated gene targeting, which was employed in a recent analysis of *dsx* function in the developing *A*. *aegypti* brain [30], was used to silence *dsx* during *A*. *aegypti* larval and/or pupal development. For larval silencing experiments, *dsx-KD A*, *dsx-KD B*, or control chitosan/siRNA nanoparticles were fed to larvae [34]. Silencing of *dsx* in chitosan/siRNA nanoparticle-fed or siRNA-injected animals was confirmed through in situ hybridization experiments. Quantification of *dsx* signal through mean gray value analyses in control vs. *dsx*-silenced animal tissues indicated that significant knockdown levels were achieved (S1 Fig). A summary of phenotypes assessed in this study and the delivery method used for analysis of each phenotype is provided in S1 Table. For analysis of structures/traits that develop in the late pupal stage, *dsx-KD A or dsx-KD B* siRNA was delivered through pupal microinjection, which generates more effective silencing at the late pupal stage, at which time some recovery of *dsx* expression is observed in animals that were fed chitosan/siRNA nanoparticles as larvae (S1 Fig).

Chitosan/siRNA larval *dsx* targeting experiments did not impact animal survival to adulthood (Table 1, n = 50 per control or experimental condition; four replicate experiments performed, P>0.05) nor impact our ability to distinguish male and female animals on the basis of their external morphology. However, as discussed further below, this chitosan/siRNA silencing of *dsx* in *A*. *aegypti* larvae led to morphological defects in the wing, proboscis, maxillary palp, and antenna. For analysis of structures/traits that develop in the late pupal stage, *dsx-KD A*, *dsx-KD B*, or control siRNAs were microinjected into female pupae [30]. As with the larval targeting experiments, survival to adulthood was not impacted in these microinjection *dsx* targeting experiments (n = 50 per control or experimental condition; three replicate experiments performed, P>0.05), and the mosquitoes could still be identified as females on the basis of the external morphology. However, as detailed herein, fertility and fecundity defects that correlated with ovary defects were observed in these animals. Olfactory phenotypes were also detected, and female lifespan decreased.

**Table 1:** Adult survival following larval targeting of *dsx*.

	Replicate-I			Replicate-II			Replicate-III			Replicate-IV		
siRNA	n	Female	Male	n	Female	Male	n	Female	Male	n	Female	Male
**Control**	50	29 (58%)	21 (42%)	50	16 (32%)	34 (68%)	50	22 (44%)	28 (56%)	50	20 (40%)	30 (60%)
***dsx*-KD A**	50	23 (46%)	27 (54%)	50	23 (46%)	27 (54%)	50	19 (38%)	31 (62%)	50	36 (72%)	14 (28%)
***dsx*-KD B**	50	24 (48%)	26 (52%)	50	26 (52%)	24 (48%)	50	27 (54%)	23 (46%)	50	27 (54%)	23 (46%)

Shown are the numbers and percentages of adult females and males that were observed following chitosan/siRNA feedings of 50 animals with control, *dsx*-KD A, or *dsx*-KD B siRNA in four replicate experiments (I-IV). No significant differences were observed in the percentages of adults, adult females, or adult males surviving following each treatment.

### Female appendage size decreases following developmental silencing of *dsx*

**Fig 1:**
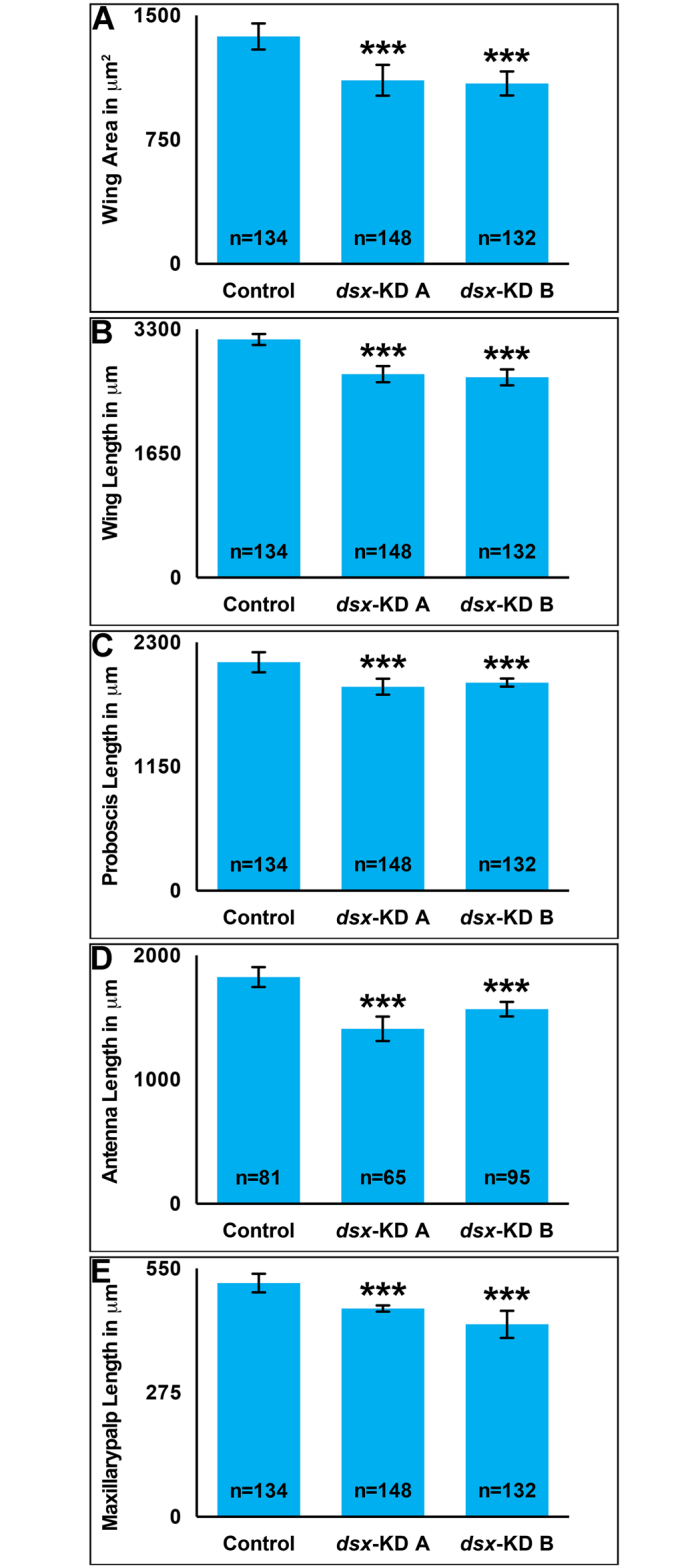
Developmental silencing of *dsx* decreases adult female appendage size. With respect to adult females fed control siRNA nanoparticles as larvae, mean adult wing areas (A), as well as mean adult wing (B), proboscis (C), antenna (D), and maxillary palp (E) lengths are significantly decreased in females fed *dsx-KD A* or *dsx-KD B* siRNA nanoparticles as larvae. Means for each control or experimental group are reported, and these were determined through the combination of data gathered in at least four biological replicate experiments. Error bars represent standard deviations. *** = P < 0.001.

*A*. *aegypti* body size, which is larger in females, can be assessed through analysis of wing size, a proxy for adult body size [46, 47]. Wing areas (p<0.0001, Fig 1A) and lengths (p<0.0001, Fig 1B) are significantly decreased in females fed *dsx-KD A* or *dsx-KD B* siRNA nanoparticles as larvae. On average, the areas of *dsx-KD A* and *dsx-KD B* wings are 19% and 21% smaller than control-fed animal wing areas, respectively (Fig 1A). Likewise, wing lengths of *dsx-KD A* animals are 15% smaller than control-fed females, while the lengths of *dsx-KD B* adult females are reduced by 16% (Fig 1B).

Adult females also have an elongated proboscis that is critical for blood feeding. In comparison to control-fed animals, proboscis length is significantly reduced in adults fed with *dsx-KD A* (P<0.00001, Fig 1C) or *dsx-KD B* (P<0.00001, Fig 1C) nanoparticles as larvae. Proboscis lengths of *dsx-KD A* females are 11% smaller than control-fed females, while the lengths of *dsx-KD B* adult females are reduced by 18%. Lengths of the antenna (Fig 1D) and maxillary palp (Fig 1E) were also assessed, and both are significantly reduced in females fed with *dsx-KD A* or *dsx-KD B* nanoparticles (P<0.0001). The antennae of animals fed with *dsx-KD A* nanoparticles are 23% shorter than those of control-fed animals, while those fed *dsx-KD B* nanoparticles are reduced by 14% (Fig 1D). The maxillary palp of *dsk-KD A* animals is 11% reduced in length with respect to the control, while maxillary palp length is 18% shorter in *dsx-KD B* females (Fig 1E).

### Targeting *dsx* results in female reproductive phenotypes

**Fig 2:**
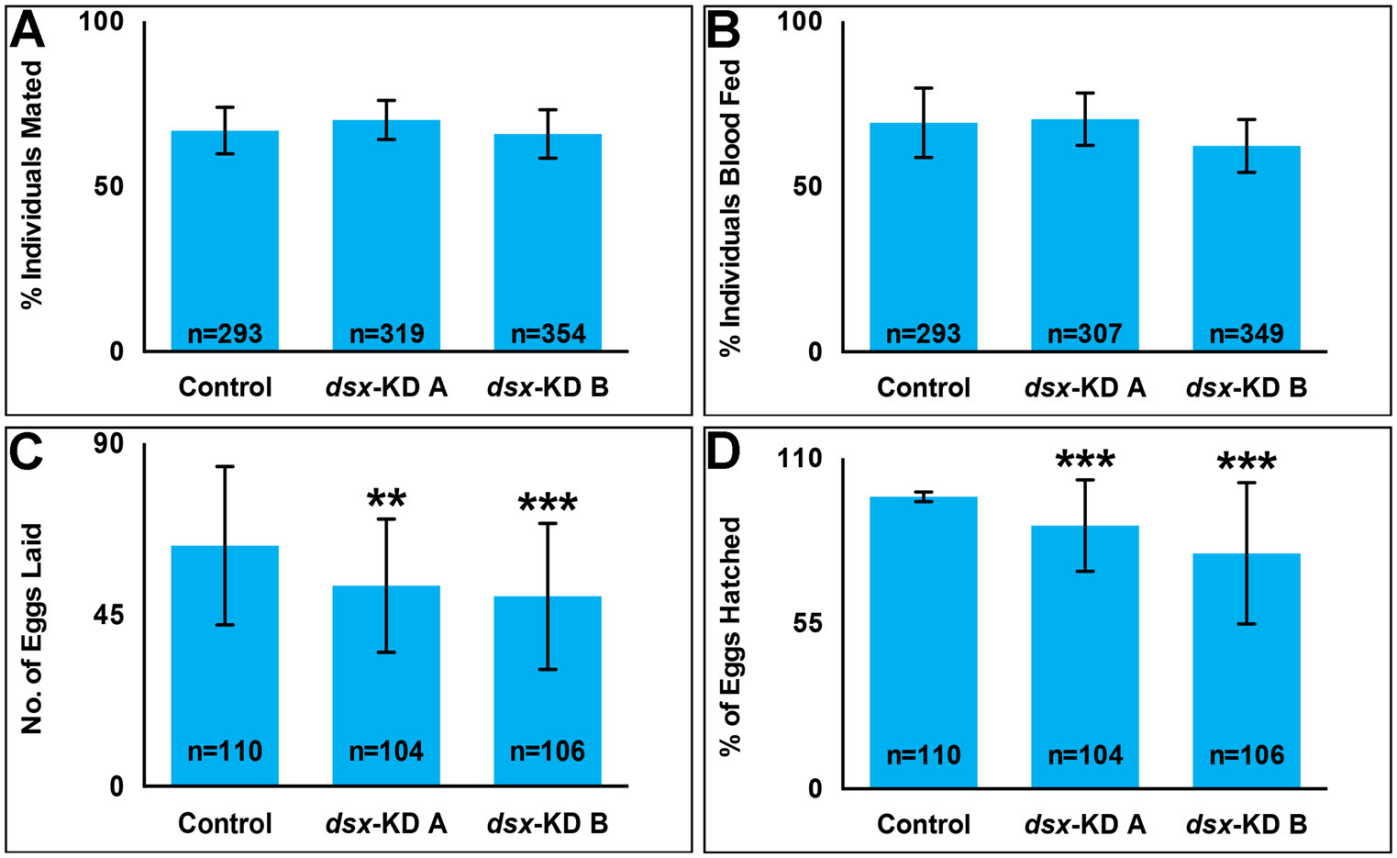
Decreased female fertility and fecundity is observed following developmental silencing of *dsx*. Although the percentages of *dsx-KD A*, *dsx-KD B*, and control siRNA-microinjected adult females that mated (A) or took blood meals (B) did not significantly differ, adult females that were injected with *dsx-KD A* or *dsx-KD B* siRNA as pupae had decreased fecundity (C) and fertility (D). Means for each control or experimental group are reported, and these were determined through the combination of data gathered in at least three biological replicate experiments. Error bars represent standard deviations.** = P < 0.01; *** = P < 0.001.

Fertility and fecundity were assessed in *dsx* KD vs. control *A*. *aegypti* females mated to wild type male mosquitoes. The number of eggs laid per female (fecundity) is significantly reduced (P<0.0001) in adult females that were injected as pupae with *dsx-KD A* or *dsx-KD B* siRNA as compared to control-injected females (Fig 2C). On average, females injected with *dsx-KD A* as pupae lay 16% fewer eggs than control-injected females, while those injected with *dsx-KD B* lay 21% fewer eggs (Fig 2C). Likewise, fertility (the percentage of hatched eggs) is significantly reduced (P<0.0001) in adult females that had been injected as pupae with *dsx-KD A* or *dsx-KD B* siRNA (Fig 2D). On average, the fertility of females injected with *dsx-KD A* or *dsx-KD B* females is 9% and 19% less, respectively, than that of control-injected females (Fig 2D).

**Fig 3:**
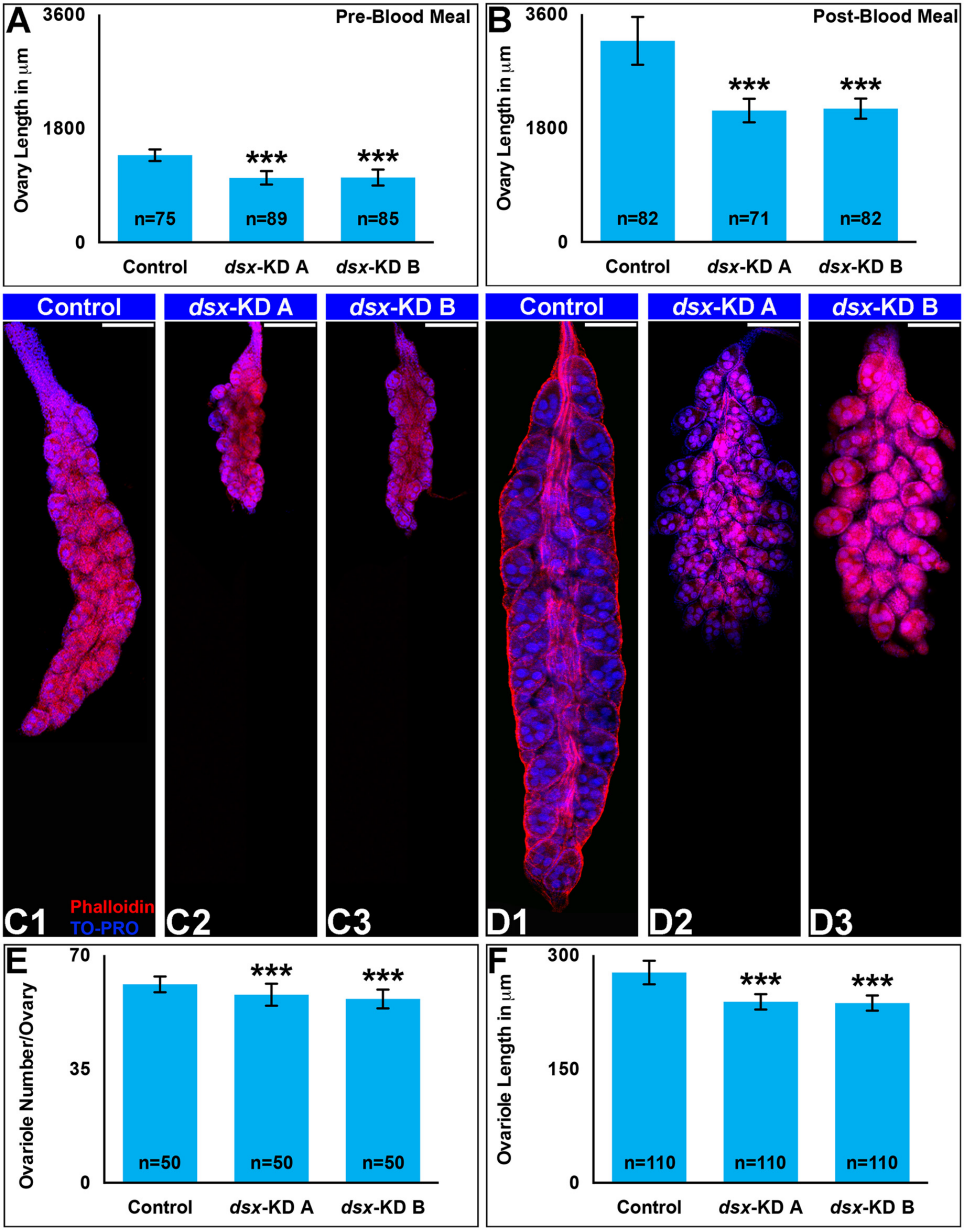
Multiple ovary phenotypes are observed following developmental silencing of *dsx*. Adult females that were injected with *dsx-KD A* or *dsx-KD B* siRNA as pupae had decreased ovary length both pre- (A) and post-blood meals (B). Representative ovaries from control-, *dsx-KD A*, or *dsx-KD B* stained with phalloidin (red) and the nuclear marker TO-PRO-3 (blue) are shown below each graph in A and B. In comparison to control microinjected females, the number of ovarioles/ovary (E) and length of each ovariole (F) post-blood meal was significantly less in females microinjected with *dsx-KD A* and *dsx-KD B* siRNA. Means for each control or experimental group are reported, and these were determined through the combination of data gathered in at least three biological replicate experiments. Error bars represent standard deviations. *** = P < 0.001.

To explore the cause of this reduced fertility and fecundity, mating, blood feeding behavior, and ovary histology were assessed in *dsx*-targeted females. In comparison to control females, no significant differences (P>0.05) were observed in the percentages of females that took blood meals (Fig 2A) or mated (Fig 2B) following larval nanoparticle or pupal microinjection delivery of *dsx-KD A* or *dsx-KD B* siRNA. Pre-blood meal ovaries were assessed at 4 days post eclosion, at which time the follicles are arrested until the mosquito takes a blood meal [48]. These experiments revealed defects in animals fed *dsx-KD A* or *B* siRNAs that were confirmed and quantified in females microinjected with *dsx-*targeting siRNAs as pupae (Fig 3). The length of pre-blood meal ovaries in *dsx-KD A* and *dsx-KD B* animals is significantly less than that of control-injected females (P<0.0001, Fig 3A, 3C1, 3C2 and 3C3). On average, the length of both *dsx-KD A* and *dsx-KD B* female ovaries is 26% shorter than control ovaries (Fig 3A). Five days post-blood meal, ovary length in 10–12 day old females that had been injected with *dsx-KD A* and *dsx-KD B* siRNA as pupae is also significantly less than that of control-injected females (P<0.0001, Fig 3B, 3D1, 3D2 and 3D3). On average, the length of *dsx-KD A* post-blood meal ovaries is 35% less than that of control ovaries, while *dsx-KD B* female ovaries are an average 34% shorter in length than control ovaries following blood meals (Fig 3B; P<0.0001). The number of ovarioles per ovary (Fig 3E) and ovariole length (Fig 3F) are also significantly reduced in post-blood meal ovaries following pupal injection with *dsx-KD A* or *dsx-KD B* siRNA (P<0.001).

### Female olfactory system defects result following targeting of *dsx*

**Fig 4:**
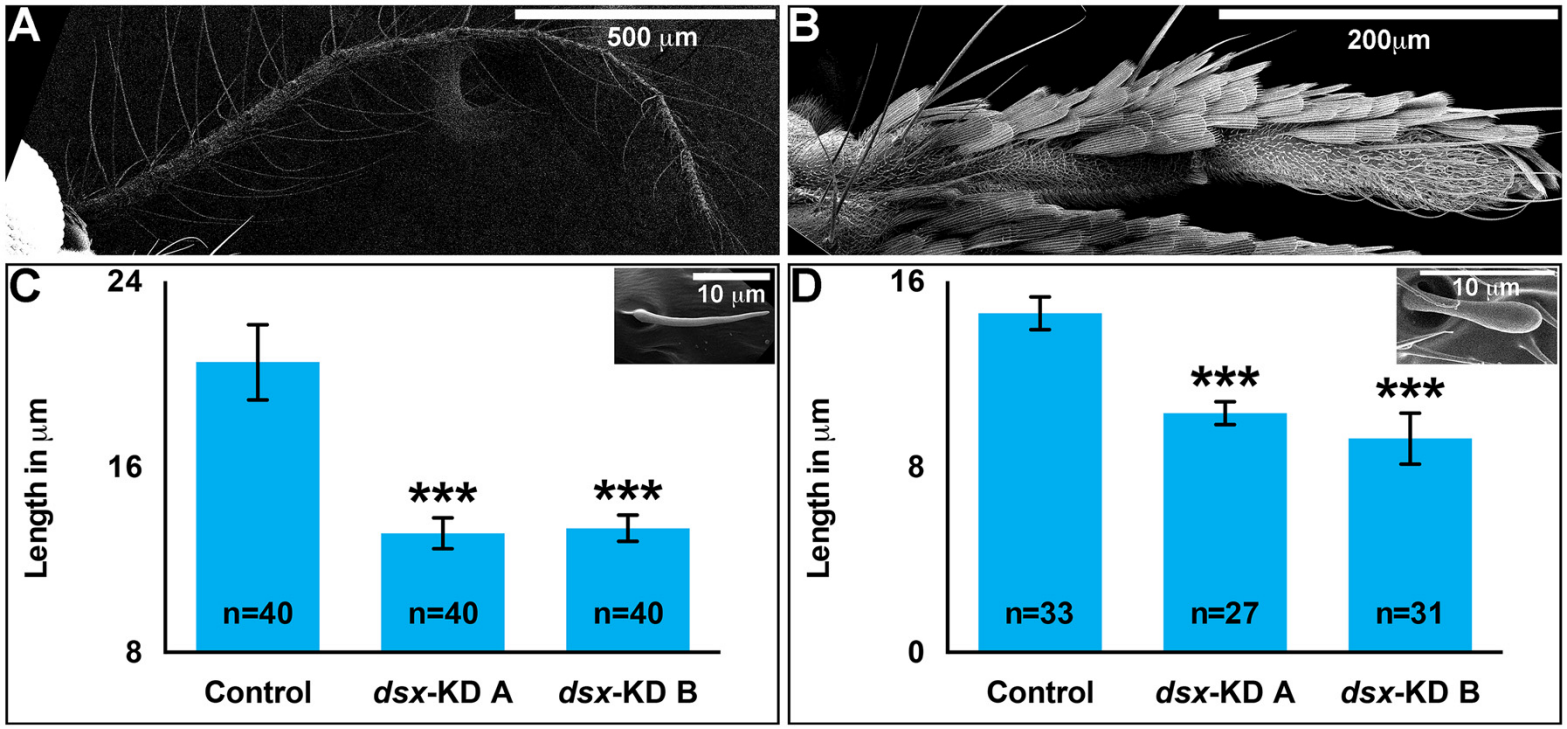
Sensillar defects result from silencing of *dsx*. Antennal *sTrichodea* (inset in C) are found on the adult female *A*. *aegypti* antenna (control-injected animal shown in A), while *sBasiconica* (inset in D) are observed on the adult female maxillary palp (control-injected animal shown in B). With respect to control-injected adult females, adult females that were injected with *dsx-KD A* or *dsx-KD B* siRNA as pupae had significantly shorter antennal *sTrichodea* (C) and maxillary palp *sBasiconica* (D). Mean sensillar lengths for each control or experimental condition are reported, and these were determined through the combination of data gathered in at least two biological replicate experiments. Error bars represent standard deviations. *** = P < 0.001.

Given the significantly shorter lengths of the female antenna and maxillary palp following silencing of *dsx* (Fig 1D and 1E), the olfactory systems of these animals were assessed in more detail. The lengths of the most abundant type of antennal sensilla, *Trichoid sensilla* (*sTrichodea*) were quantified through analysis of SEM images of the adult female antenna (Fig 4A and 4C). The length of these structures in females that had been injected with *dsx-KD A* or *dsx-KD B* siRNA as pupae is significantly less than that of control-injected females (P<0.0001, Fig 4C). On average, the length of *dsx-KD A* female antennal *sTrichodea* is 36% less than that of control *sTrichodea*, while *dsx-KD B* female *sTrichodea* are 34% shorter in length than control *sTrichodea* (Fig 4C). The lengths of *Basiconic sensilla* (*sBasiconica)* were assessed through analysis of SEM images of the adult female maxillary palp (Fig 4B and 4D). Likewise, the lengths of these structures are significantly reduced in females that had been injected with *dsx-KD A* or *dsx-KD B* siRNA as pupae (P<0.0001, Fig 4D). On average, the length of *dsx-KD A* female *sBasiconica* is 29% less than that of control *sBasiconica*, while *dsx-KD B* female *sBasiconica* are 37% shorter in length (Fig 4D).

**Fig 5:**
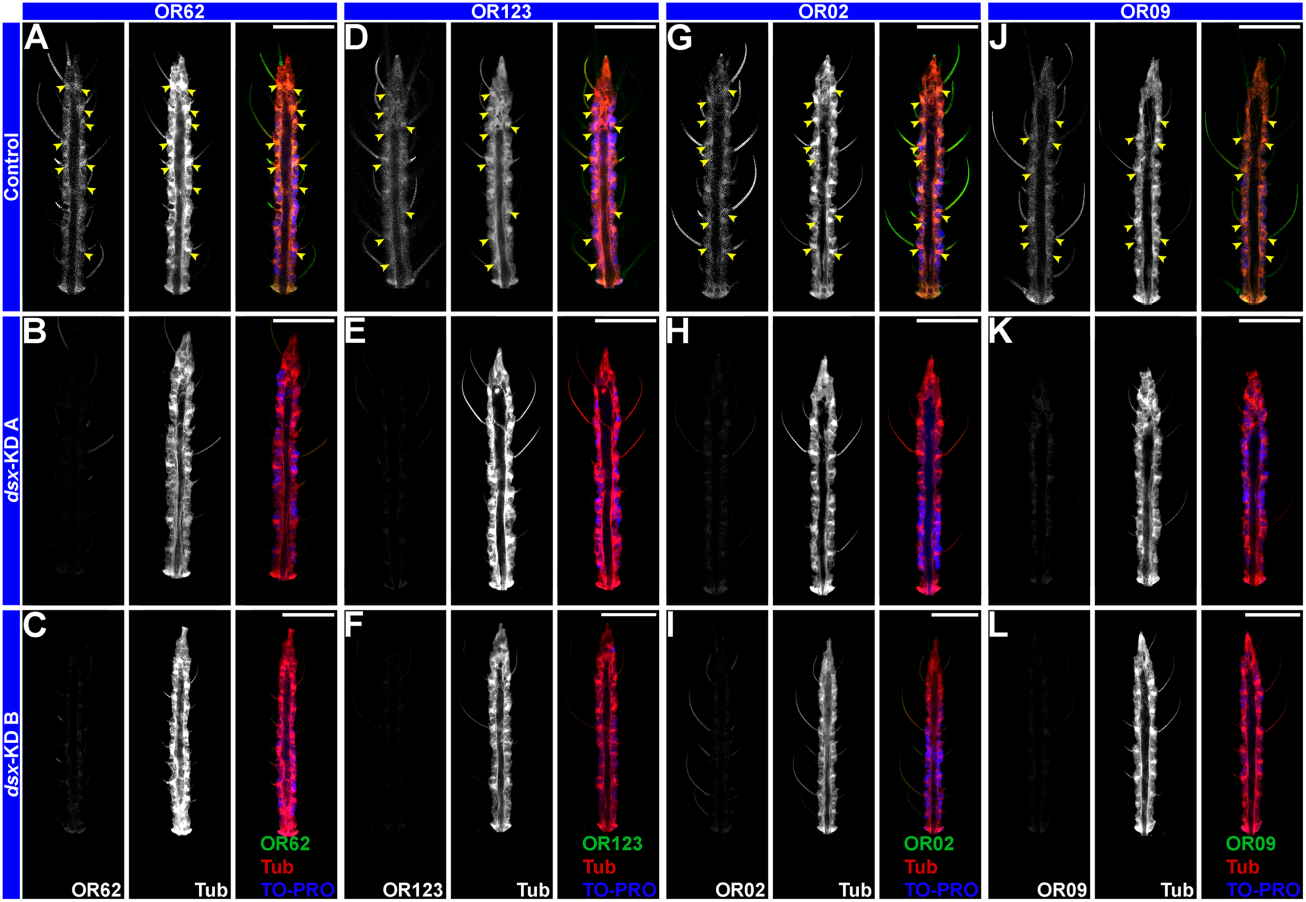
Dsx regulates *OR* expression. Terminal segments of adult female antennae oriented distal upward that were hybridized with *OR 62* (A, B, C), *OR 123* (D, E, F), *OR 2* (G, H, I), or *OR 9* (J, K, L) riboprobes (green, at left in each panel) are shown. Antennae were counter-stained with anti-Tubulin (red, center of each panel) and TO-PRO-3 nuclear stain (blue in overlays at right in each panel). *OR 62* (A), *OR 123* (D), *OR 2* (G), and *OR 9* transcripts are detected in ORNs of control-injected adult female antennae (A, D, G, J). Little expression of these transcripts could be detected in adult females injected with *dsx-KD A* (B, E, H, K) or *dsx-KD B* (C, F, I, J) siRNA as pupae. Representative results from a total of 24 antennae stained in two separate replicate experiments are shown, and analysis of *OR* expression intensity levels is presented in S3 Table. Scale bars = 25 microns. Arrowheads mark ORNs.

Expression of several *odorant receptor (OR)* genes was assessed in *A*. *aegypti* adults. Bohbot et al. [49] reported female-specific expression of *OR 62* and *OR 123* that was detected through qRT-PCR studies. WhoIe-mount *in situ* hybridization detected expression of *OR 62* (Fig 5A) and *OR 123* (Fig 5D) transcripts in the antennae of adult females. Sequences that match the Dsx consensus binding site sequence [45] were identified upstream of the *OR 62* and *OR 123* open reading frames (S2 Table). Although it was not biochemically assessed whether Dsx can bind to these sequences, expression of both *OR* genes is disrupted in the antennae of adult females that had been microinjected with *dsx-KD A* (Fig 5B and 5E) or *dsx-KD B* siRNA (Fig 5 and 5F) as pupae. Mean gray scale analyses indicated that the *OR 62* and *OR 123* transcript signals in the antennae of *dsx-KD A* and *dsx-KD B* animals were significantly reduced in comparison to those of control-injected animals (P<0.0001; S3 Table). Likewise, sequences that match the Dsx consensus binding site sequence [45] were identified upstream of the *OR 2* and *OR 9* open reading frames (S2 Table). Adult female expression of both genes (Fig 5G and 5J) is disrupted in the antennae of females injected with *dsx-KD A* (Fig 5H and 5K) or *dsx-KD B* (Fig 5I and 5J) as pupae. Mean gray scale analyses for these expressions studies demonstrated that the *OR 2* and *OR 9* transcript signals in the antennae of *dsx-KD A* and *dsx-KD B* animals were also significantly reduced in comparison to those of control-injected animals (P<0.0001; S3 Table). Thus, *dsx* silencing resulted in multiple *OR* expression defects in adult female mosquitoes.

### Developmental targeting of *dsx* results in decreased female lifespan

**Fig 6:**
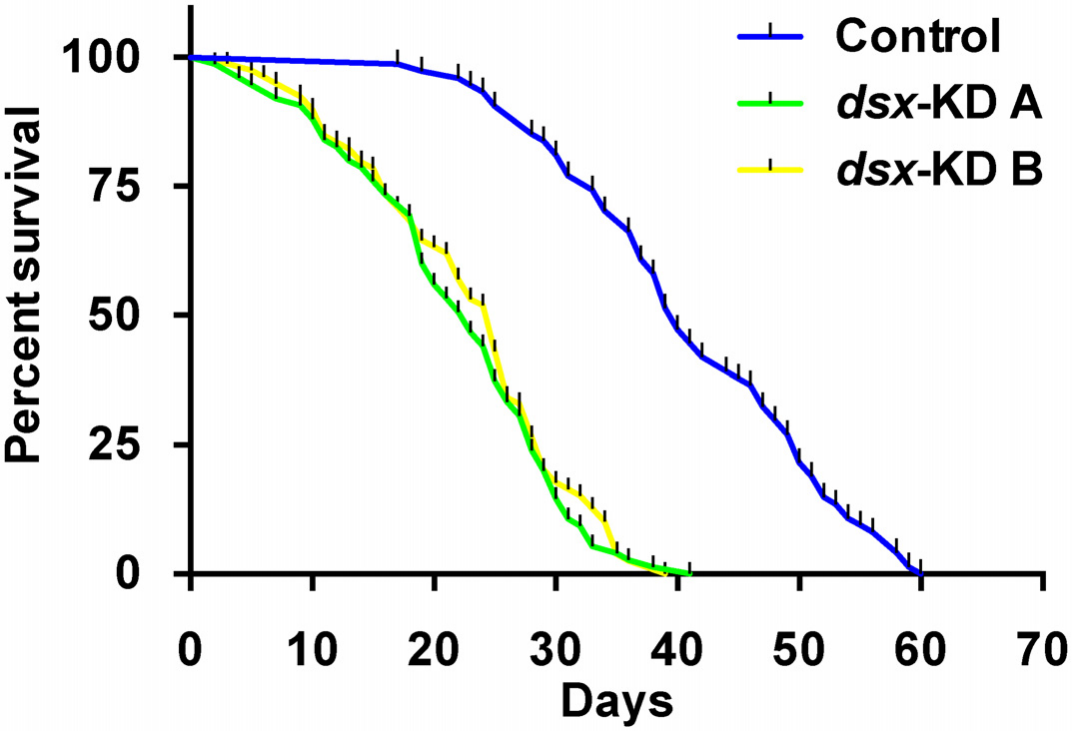
Adult female lifespan decreases following developmental silencing of *dsx*. Survivorship curves for animals injected as pupae with control, *dsx-KD A*, or *dsx-KD B* siRNA are shown. Analysis of data combined from three replicate experiments indicated that in comparison to the control, life span was significantly decreased in individuals injected with *dsx-KD A* (P<0.001) or *dsx-KD B* (P<0.001) siRNA.

The impact of developmental targeting of *dsx* on adult female survival was also assessed. The median survival rates for animals injected with control, *dsx-KD A*, and *dsx-KD B* siRNA were found to be 40, 23, and 25 days, respectively. In comparison to control-injected animals, life span was significantly decreased in individuals injected with *dsx-KD A* (P<0.001) or *dsx-KD B* (P<0.001) siRNA (Fig 6). These data demonstrate that silencing *dsx* during pupal development results in a significantly shorter female adult lifespan.

## Discussion

Despite the long-standing interest in genes that regulate sex determination and sex differentiation in mosquitoes, functional genetic characterization of such loci has been a challenge. Here, siRNA-mediated gene silencing was applied for successful functional characterization of *A*. *aegypti dsx*, a terminal transcription factor in the insect sex determination pathway, during development of female mosquitoes. The adult phenotypes examined fell into four general categories that are discussed in more detail below: growth (Figs 1 and 3), reproduction (Figs 2 and 3), olfactory (Figs 4 and 5), and life span (Fig 6).

### Impacts of Dsx silencing on growth

Body size is a sexually dimorphic trait in *A*. *aegypti*, in which females are larger than males [37]. Loss of *dsx* function resulted in significantly smaller wing size, a correlate for body size (Fig 1A and 1B). The impact of *dsx* silencing was observed in a number of other tissues, including the proboscis, antenna, maxillary palp (Fig 1C,1D, 1E and 1F), ovaries (Fig 3) and sensilla (Fig 4), all of which were significantly smaller with respect to control animals. Although one might have expected to observe a longer male-like maxillary palp in *dsx*-silenced adult females, this was not found to be the case. Interestingly, our preliminary data suggest that much like females, silencing of *dsx* in male mosquitoes results in decreased body size and appendage lengths. These findings suggest that Dsx may function in *A*. *aegypti* to positively regulate growth of both sexes. Dsx has been associated with growth in a number of other insects and in additional tissue types. For example, in a study of *Nasonia* species, Loehlin et al. [50] found that wing size reduction correlated with an increase in *dsx* wing expression levels that is specific to developing males of this species. Sex-specific Dsx splice forms are known to regulate sexually dimorphic exaggerated male horn development in two species of beetles, *O*. *taurus* [18] and the rhinoceros beetle *T*. *dichotomus* [19]. In male *O*. *taurus*, silencing of *dsx* reduced horn development in large males, while silencing of *dsx* in females resulted in induction of ectopic, nutrition-sensitive horn development in females that are otherwise hornless. Comparable results were obtained in *T*. *dichotomus* [19]. Dsx also regulates sex-specific mandible growth, which is exaggerated in *Cyclommatus metallifer* males [21].

Our recent study demonstrated that genes linked to the cell cycle are upregulated in *A*. *aegypti* females [30]. These genes, which are likely associated with increased growth of female tissues, may be direct or indirect targets of Dsx. In support of this notion, *cyclin dependent kinase 4/6 (cdk4/6)*, a positive regulator of cellular growth in *D*. *melanogaster* [51, 52], is upregulated in the *A*. *aegypti* female pupal brain. Dsx consensus binding sites flank the *A*. *aegypti cdk 4/6* gene, and sexually dimorphic expression of this gene in the pupal brain is disrupted by *dsx* knockdown [30]. Interestingly, Cyclin D, the Cyclin associated with Cdk4/6 in *D*. *melanogaster* [53], was recently identified as a Dsx target gene in *Drosophila* [45]. It is therefore likely that loss of Cyclin D-Cdk4/6 function may be at least in part responsible for the size differences observed upon silencing of *dsx* in both sexes of *A*. *aegypti*. Additionally, Gotoh et al. [21] identified a link between *dsx* and the juvenile hormone (JH) signaling pathway, demonstrating that female-specific splice variants of *C*. *metallifer dsx* contribute to the insensitivity of female mandibles to JH. It would therefore be interesting to determine if sex-specific Dsx splice forms regulate JH-responsiveness in *A*. *aegypti*.

### Impacts of Dsx targeting on reproduction

Loss of Dsx function results in sterility in *D*. *melanogaster* [4] and *Bombyx mori* [20], and RNAi targeting of *dsx* during pupal development in *T*. *casteneum* was found to result in decreased fertility and fecundity [17]. Decreased female fertility and fecundity were observed when *dsx* was silenced during female *A*. *aegypti* pupal development (Fig 2). These differences correlated with decreased adult ovary length both pre- and post-blood meal, as well as a decreased ovariole length and number in blood-fed adult females (Fig 3). In *D*. *melanogaster*, Dsx was found to play early roles in the development of the female/male genitalia and analia, which are both derived from the larval genital imaginal disc. *D*. *melanogaster* Dsx regulates the anterior/posterior organizer to control growth of female or male genital primordia, and then it acts in a sex-specific manner to direct differentiation of each male or female primordium into the defined adult structures present in either sex [54]. Dsx may have similar roles in *A*. *aegypti*, and so it would be interesting to assess its function at the level of the genital primordia, the development of which has not yet been assessed. Here, we focused on later stages of ovary development, which in mosquitoes is dependent upon the acquisition of a blood meal, and which has been better characterized [48].

Vitellogenesis, the synthesis and secretion of yolk protein precursors (YPPs), is a critical event in the mosquito reproductive cycle that is activated in response to the blood meal [55, 56]. In *D*. *melanogaster*, which does not require a blood meal for reproduction, the vitellogenin subunit *yolk protein-1 (yp1)* and *yp2* genes are targets of Dsx [57, 58]. Similarly, the *T*. *casteneum* YPP *vitellogenin (vg)* and *vitellogenin receptor (vgr)* genes were identified as targets of *Tc* Dsx [17]. Thus, one might speculate that Dsx regulation of *YPP* genes is conserved in insects. However, the *A*. *aegypti* YPP genes lack any obvious Dsx binding sites, and if Dsx regulates expression of these genes in *A*. *aegypti*, it is likely to do so indirectly. Interestingly, juvenile hormone (JH) is known to regulate expression of *A*. *aegypti* Vg-A [59]. Although we were unable to identify any obvious Dsx consensus binding site sequences associated with components of the JH signaling pathway in *A*. *aegypti*, Gotoh *et al*. [21] recently demonstrated that female-specific splice variants of *C*. *metallifer dsx* contribute to the insensitivity of female mandibles to JH. It would therefore be interesting to determine if links between Dsx and JH responsiveness exist in *A*. *aegypti*.

Dsx likely regulates *A*. *aegypti* female reproduction in many additional ways. Although we did not detect any significant impacts of Dsx silencing on membrane blood feeding behavior or the ability to mate in the laboratory setting (Fig 2A and 2B), it is not known how these mosquitoes would perform in the wild. Furthermore, Lee et al. [60] recently identified a Dsx-positive neuronal pathway in *D*. *melanogaster* that controls sperm ejection and storage. When the neuronal signaling pathway in the brain, which consists of Diuretic hormone 44 (Dh44) and its receptor (Dh44R1), is suppressed, the brain expedites sperm ejection from the uterus, resulting in decreased fecundity. Thus, Dsx signaling could have multiple impacts on female reproduction in *A*. *aegypti*. Gaining a fuller understanding of these impacts will likely require much more detailed knowledge of *A*. *aegypti* reproductive behaviors.

### Impacts of Dsx on the olfactory system

This investigation has linked Dsx with the expression of *OR* genes. This linkage, which to our knowledge has yet to be identified in other organisms, provides insight into the regulation of sex-specific olfactory development. Silencing of *dsx* during pupal development was found to disrupt expression of two female *ORs*, *62* and *123* (Fig 5). Although the functions of these ORs have yet to be characterized in *A*. *aegypti*, upregulation of the expression of these genes by Dsx^F^ may contribute to female-specific olfactory-driven behaviors. The detection of Dsx consensus binding site sequences upstream of the *OR 62* and *123* open reading frames (S2 Table) suggests that their expression may be regulated directly by Dsx, but this has not yet been directly assessed. Dsx also positively regulates expression of *ORs 2* and *9* (Fig 5). The function of *A*. *aegypti OR 9* is not known. However, *OR 2*, which is well conserved among mosquitoes, is known to be activated by indole, a major volatile component of human sweat that is also implicated in oviposition site selection [61, 62].

To date, analysis of the development of sexual dimorphism in the olfactory system has largely centered on analysis of the roles of *fruitless (fru)*, which also encodes a terminal transcription factor in the sex determination pathway that is spliced in a sex-specific manner [8]. Cachero et al. [63] performed a global search for sexually dimorphic structural differences in the *Drosophila* brain, as well as a saturating clonal analysis of Fru-positive neurons. They noted that the proportion of cells in the *D*. *melanogaster* brain that expresses Dsx is smaller and partially overlaps with Fru, an interesting observation given that Neville et al. [64] suggested that *Drosophila* Dsx and Fru may act together, either in a physical complex or through co-regulation of target genes, to control sex-specific neural development. It will therefore be interesting to functionally assess the roles of Fru during neural development in *A*. *aegypti*.

*Drosophila* researchers have begun to link Dsx and Fru to specific sexually dimorphic neural physiologies, neural circuitries, and behaviors, and so another challenge will be to further develop genetic technologies with the goal of being able to perform comparably technical analyses in mosquitoes. For example, in *D*. *melanogaster*, the activation of a set of Fru-positive olfactory receptor neurons (ORNS) that express OR67d, which responds to male pheromone cis-vaccenyl acetate, was found to inhibit male courtship of other males and induce female receptivity to other males. The ORNs expressing 67d converge to a single glomerulus, DA1, in both the sexes, but the projections from the DA1 glomerulus to the protocerebrum were found to be sexually dimorphic, suggesting that differential behaviors induced by this pheromone result from sex-specific neural circuitries [65]. Kohl et al. [66] further demonstrated that sex-specific wiring induces differential responses to cVA pheromone inputs, suggesting that different *fru* isoforms function as a bidirectional switch to activate different behaviors in males and females. In another study, reduction of Ecdysone receptor-A in Fru^M^-positive neurons, which is associated with an increase in male-male courtship activity, was found to result in significant reduction in the size of two antennal lobe glomeruli, suggesting that EcR-A is required for establishment of male-specific neuronal architecture in the *D*. *melanogaster* olfactory system [67]. These findings suggest that in addition to differences in *OR* expression, changes in the overall neural circuitry responding to the odorant may induce dimorphic behaviors in males and females, and it will be interesting to examine this in *A*. *aegypti* in the future.

### Dsx targeting reduces female lifespan

As discussed by Brady et al. [68], the survival of arthropod vectors is one of the most critical components of pathogen transmission. Increased survival results in the production of more offspring. It also increases the likelihood of the arthropod to become infected, to disperse over greater distances once infected, to survive long enough to transmit the pathogen, and to deliver a greater number of infectious bites during its lifespan. Thus, small changes in survival rates could have large impacts on pathogen transmission [69–72], and vector control strategies that shorten vector lifespan may represent new alternative control strategies [73–75].

The results of this study demonstrate that targeting *dsx* during female pupal development significantly reduces adult *A*. *aegypti* female lifespan (Fig 6). The genetic manipulation of sex-determination gene expression in *D*. *melanogaster* has been shown to impact lifespan [76]. While overexpression of *dsx* ^*F*^ during male development was lethal to males and females (with a limited number of female escapers), overexpression of *dsx* ^*F*^ in adults dramatically reduced the life span of both males and females. Overexpression of the male isoform of *fru* in males or females yielded similar results. Interestingly an RNAi line targeting *fru* reduced lifespan in *D*. *melanogaster* females only. Shen et al. [76] suggested that it would be interesting to examine potential interactions between the sex determination genes and the insulin/IGF1-like signaling (IIS) pathway or dietary restriction, both of which regulate lifespan in a sex-dependent manner. Furthermore, Tarone et al. [77] demonstrated that Yp expression is negatively correlated with longevity in *D*. *melanogaster*. Thus, as discussed above, any impact that Dsx might have on Yp expression could underlie the decreased longevity of *A*. *aegypti dsx*-targeted females. Finally, silencing *dsx* in *A*. *aegypti* was shown to result in decreased expression of *p53* [30]. Overexpression of *p53* in the *D*. *melanogaster* female nervous system results in increased life span [76]. It is tempting to speculate that downregulation of *p53* expression following *dsx* silencing may contribute to the decreased lifespan observed in *dsx*-targeted *A*. *aegypti* females.

## Conclusion

Female mosquitoes differ from males in several morphological, physiological, and behavioral traits that are critical to their ability to transmit diseases. The arthropod disease vector research community has therefore had a long-standing interest in the potential to manipulate sex determination and differentiation genes for controlling disease vectors. Our previous work [30] demonstrated that Dsx regulates sex-specific gene expression in the developing *A*. *aegypti* pupal nervous system. The present investigation extended these initial findings through assessment of the effects of developmental siRNA-mediated *dsx* silencing in adult females. Targeting of *dsx* resulted in decreased size of the female wing and proboscis (Fig 1). Decreased fecundity and fertility correlated with decreased ovary length, ovariole length, and ovariole number in females in which *dsx* was silenced during development (Figs 2 and 3). Targeting *dsx* also resulted in disruption of olfactory system development, as evidenced by reduced length of the female antenna and maxillary palp and their respective sensilla (Figs 1 and 4), as well as disrupted *OR* expression (Fig 5). Female lifespan, a critical aspect of mosquito pathogen transmission, was also significantly reduced in adult females following developmental targeting of *dsx* (Fig 6). These results demonstrate that developmental silencing of *dsx* in *A*. *aegypti* females, which disrupts development of multiple adult female traits linked directly or indirectly to reproduction and pathogen transmission, may be useful for vector control.

## Supporting Information

S1 Fig Developmental silencing of *dsx*. Silencing of *dsx* was confirmed through *in situ* hybridization following pupal microinjection (A1-4, B1-4) or chitosan/siRNA nanoparticle feedings (C1-4, D1-4, E1-4) of control, *dsx-KD A*, or *dsx-KD B* siRNA. 24 hr APF brains (A1-3, C1-3) and antennae (B1-3), as well as fourth larval instar brains (D1-3) and antennae (E1-3) are shown. Corresponding mean gray values from 19 or more tissue samples (n values are noted) compiled from two replicate experiments are shown at right (A4, B4, C4, D4, E4). In comparison to control tissues, significantly lower values were detected for *dsx-KD A* and *dsx-KD B* tissues (P<0.0001***). Error bars denote standard deviations. Percentage decreases in mean gray values are indicated for *dsx-KD A* or *dsx-KD B* treatments in each chart.
(PDF)

S1 Table Method of siRNA delivery for phenotypes assessed. The method of siRNA delivery (larval chitosan nanoparticle feeding or pupal microinjection) is noted for each phenotype assessed in the investigation.
(PDF)

S2 Table Dsx consensus binding sequences upstream of *A*. *aegypti OR* genes. 5’ flanking Dsx consensus binding sequences were identified upstream of the indicated *OR* genes. The position of each sequence relative to the open reading frame of each *OR* gene is indicated.
(PDF)

S3 Table Quantifying the impact of *dsx* silencing on *OR* levels in the female antenna. Mean gray values for the indicated *OR* transcript signals detected in 24 adult female antennae (compiled from two replicate experiments) following pupal microinjection of control, *dsx-KD A*, or *dsx-KD B* siRNA. Significantly lower values were detected for *dsx-KD A* or *dsx-KD B* vs. control animals (P<0.0001***). SD = standard deviation.
(PDF)
