# Supplementary material for: siRNA-Mediated Silencing of doublesex during Female Development of the Dengue Vector Mosquito Aedes aegypti
Source: PLoS Negl Trop Dis. 2015 Nov 6;9(11):e0004213. doi: 10.1371/journal.pntd.0004213 (PMC4636264; doi:10.1371/journal.pntd.0004213)
Supplement: S1 Fig — Silencing of dsx was confirmed through in situ hybridization following pupal microinjection (A1-4, B1-4) or chitosan/siRNA nanoparticle feedings (C1-4, D1-4, E1-4) of control, dsx-KD A, or dsx-KD B siRNA. 24 hr APF brains (A1-3, C1-3) and antennae (B1-3), as well as fourth larval instar brains (D1-3) and antennae (E1-3) are shown. Corresponding mean gray values from 19 or more tissue samples (n values are noted) compiled from two replicate experiments are shown at right (A4, B4, C4, D4, E4). In comparison to control tissues, significantly lower values were detected for dsx-KD A and dsx-KD B tissues (P<0.0001***). Error bars denote standard deviations. Percentage decreases in mean gray values are indicated for dsx-KD A or dsx-KD B treatments in each chart. (PDF) [file pntd.0004213.s001.pdf]

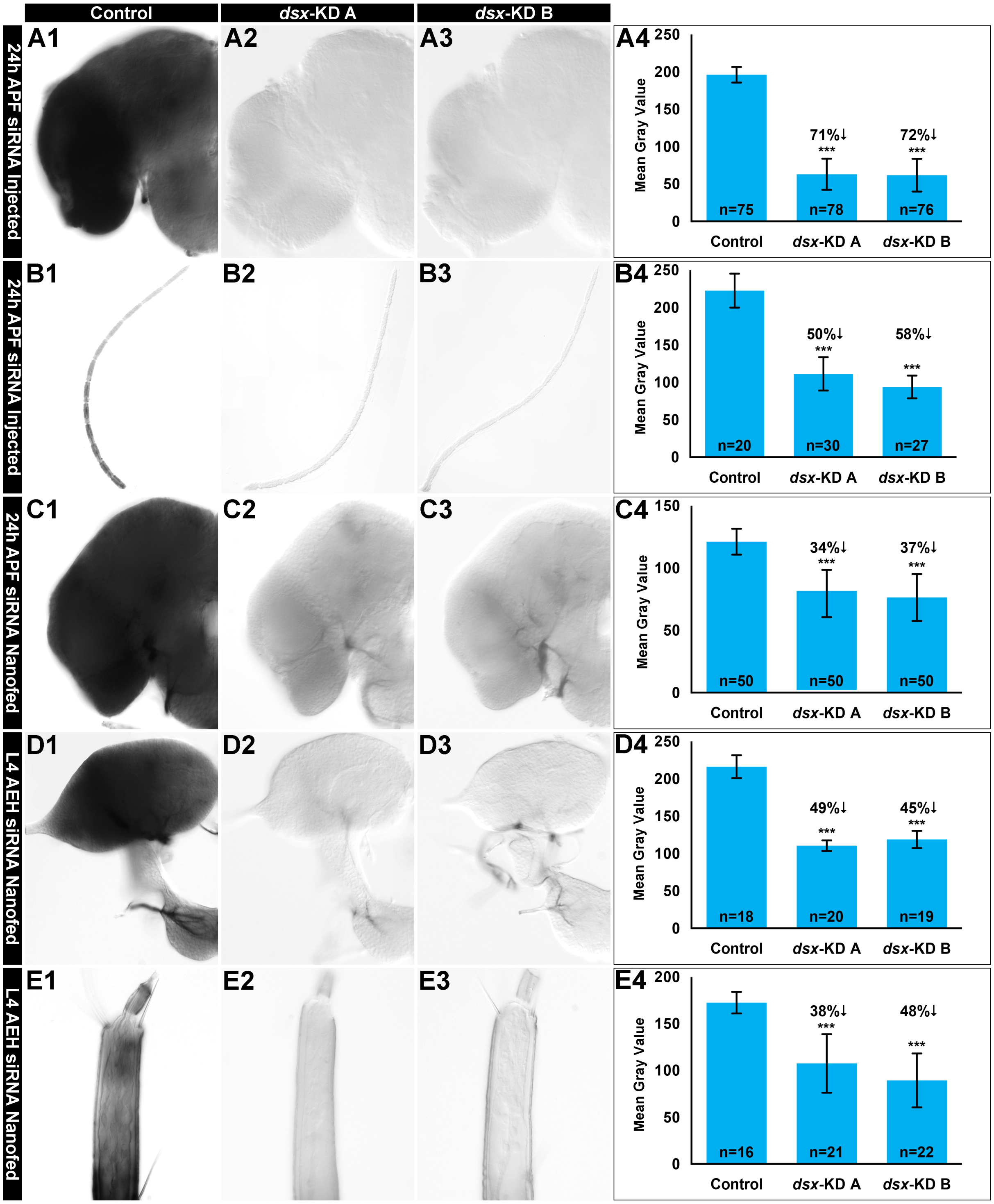

S1 Figure. Developmental silencing of *dsx*. Silencing of *dsx* was confirmed through *in situ* hybridization following pupal microinjection (A1-4, B1-4) or chitosan/siRNA nanoparticle feedings (C1-4, D1-4, E1-4) of control, *dsx-KD A,* or *dsx-KD B* siRNA. 24 hr APF brains (A1-3, C1-3) and antennae (B1-3), as well as fourth larval instar brains (D1-3) and antennae (E1-3) are shown. Corresponding mean gray values from 19 or more tissue samples (n values are noted) compiled from two replicate experiments are shown at right (A4, B4, C4, D4, E4). In comparison to control tissues, significantly lower values were detected for *dsx-KD A* and *dsx-KD* tissues (P<0.0001***). Error bars denote standard deviations. Percentage decreases in mean gray values are indicated for *dsx-KD A* or *dsx-KD B* treatments in each chart.
