# Supplementary material for: siRNA-Mediated Silencing of doublesex during Female Development of the Dengue Vector Mosquito Aedes aegypti
Source: PLoS Negl Trop Dis. 2015 Nov 6;9(11):e0004213. doi: 10.1371/journal.pntd.0004213 (PMC4636264; doi:10.1371/journal.pntd.0004213)
Supplement: S1 Table — The method of siRNA delivery (larval chitosan nanoparticle feeding or pupal microinjection) is noted for each phenotype assessed in the investigation. (PDF) [file pntd.0004213.s002.pdf]

S1 Table. Method of siRNA delivery for phenotypes assessed

Phenotype	siRNA delivery method	
Wing Area	Chitosan nanoparticle	-
Wing Length	Chitosan nanoparticle	-
Proboscis Length	Chitosan nanoparticle	-
Antenna Length	Chitosan nanoparticle	-
Maxillary palp Length	Chitosan nanoparticle	-
Fertility and Fecundity	-	Pupal Microinjection
Ovary Length (pre-bloodmeal)	-	Pupal Microinjection
Ovary Length (post-bloodmeal)	-	Pupal Microinjection
Ovariole Number	-	Pupal Microinjection
Ovariole Length	-	Pupal Microinjection
Sensilla Length	-	Pupal Microinjection
OR expression	-	Pupal Microinjection
Longevity	-	Pupal Microinjection

The method of siRNA delivery (larval chitosan nanoparticle feeding or pupal microinjection) is noted for each phenotype assessed in the investigation.
