# Supplementary material for: siRNA-Mediated Silencing of doublesex during Female Development of the Dengue Vector Mosquito Aedes aegypti
Source: PLoS Negl Trop Dis. 2015 Nov 6;9(11):e0004213. doi: 10.1371/journal.pntd.0004213 (PMC4636264; doi:10.1371/journal.pntd.0004213)
Supplement: S2 Table — 5’ flanking Dsx consensus binding sequences were identified upstream of the indicated OR genes. The position of each sequence relative to the open reading frame of each OR gene is indicated. (PDF) [file pntd.0004213.s003.pdf]

S2 Table. Dsx consensus binding sequences upstream of *A. aegypti OR* genes

***Aae* Gene ID**	**Gene Name**	**Sequence**	**Position**
AAEL005999	*OR 2*	AAAACAAAGATGG	2 kb upstream
		CGAACAATGTTGT	2.5 kb upstream
		ATTACTAAGAAAT	3.3 kb upstream
		ATGACAATGTAAT	2.7 kb upstream
AAEL006005	*OR 9*	ATGACTATGTCGT	2.9 kb upstream
		GCAAATATGTTGC	3.2 kb upstream
		CACACTAAGTTAT	3.4 kb upstream
AAEL011796	*OR 62*	CCTACAAAGAATC	0.7 kb upstream
		ACTACAATGAATT	0.9 kb upstream
		GACACTAAGTTTC	3.6 kb upstream
		CATAAAATGTTGA	0.9 kb upstream
AAEL017537	*OR 123*	GTAACAAAGTTTA	1.8 kb upstream
		AAAACAAAGTTTG	4.0 kb upstream

5' flanking Dsx consensus binding sequences were identified upstream of the indicated *OR* genes. The position of each sequence relative to the open reading frame of each *OR* gene is indicated.
