# Supplementary material for: siRNA-Mediated Silencing of doublesex during Female Development of the Dengue Vector Mosquito Aedes aegypti
Source: PLoS Negl Trop Dis. 2015 Nov 6;9(11):e0004213. doi: 10.1371/journal.pntd.0004213 (PMC4636264; doi:10.1371/journal.pntd.0004213)
Supplement: S3 Table — Mean gray values for the indicated OR transcript signals detected in 24 adult female antennae (compiled from two replicate experiments) following pupal microinjection of control, dsx-KD A, or dsx-KD B siRNA. Significantly lower values were detected for dsx-KD A or dsx-KD B vs. control animals (P<0.0001***). SD = standard deviation. (PDF) [file pntd.0004213.s004.pdf]

S3 Table. Quantifying the impact of *dsx* silencing on *OR* levels in the female antenna

**Transcript**	***OR 2***		***OR 9***		***OR 2***		***OR 123***	
	**Mean Gray Value**	**SD**	**Mean Gray Value**	**SD**	**Mean Gray Value**	**SD**	**Mean Gray Value**	**SD**
**Control**	123.31	5.8	132.63	4.9	125.88	10.2	109.17	5.0
dsx-KD A	68.55***	19.2	72.38***	29.8	74.50***	26.4	65.76***	19.9
dsx-KD B	66.50***	17.7	68.71***	28.5	69.45***	24.9	71.30***	23.1

Mean gray values for the indicated *OR* transcript signals detected in 24 adult female antennae (compiled from two replicate experiments) following pupal microinjection of control, *dsx-KD A,* or *dsx-KD B* siRNA. ***Significantly lower values were detected for *dsx-KD A* or *dsx-KD B* vs. control animals (P<0.0001). SD = standard deviation.
